# Supplementary material for: NLRP3 Inflammasome Involved with Viral Replication in Cytopathic NADL BVDV Infection and IFI16 Inflammasome Connected with IL-1β Release in Non-Cytopathic NY-1 BVDV Infection in Bovine Macrophages
Source: Viruses. 2023 Jun 30;15(7):1494. doi: 10.3390/v15071494 (PMC10386432; doi:10.3390/v15071494)
Supplement: Supplementary file 1 [file viruses-15-01494-s001.zip › viruses-2403530-supplementary.pdf]

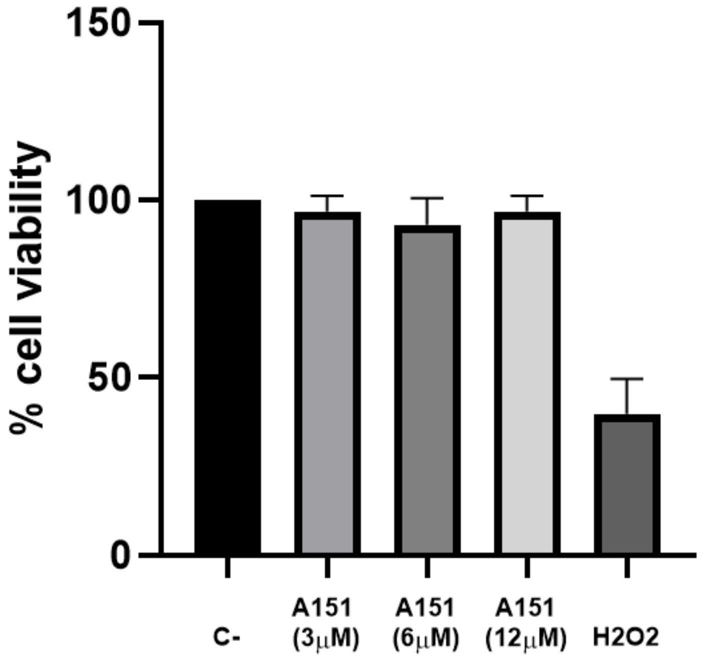

Supplementary Figure S1. Cell viability of bovine macrophages incubated with different concentration of ODN A151 inhibitor (5′-TTAGGGTTAGGGTTAGGGTTAGGG-3′) (3μM, 6μM and 12μM). Negative control: Bovine macrophages cultures with RPMI 10%FBS. Positive control: Bovine macrophages incubated with hydrogen peroxide.
